# Supplementary material for: Association between oral microbiome and seven types of cancers in East Asian population: a two-sample Mendelian randomization analysis
Source: Front Mol Biosci. 2023 Nov 21;10:1327893. doi: 10.3389/fmolb.2023.1327893 (PMC10702768; doi:10.3389/fmolb.2023.1327893)
Supplement: Supplementary file 1 [file DataSheet1.ZIP › Supplementary Materials/MR plots for tongue/tongue═╝/Prostate cancer/pheno.2411_to_prostate cancer_forest.pdf]

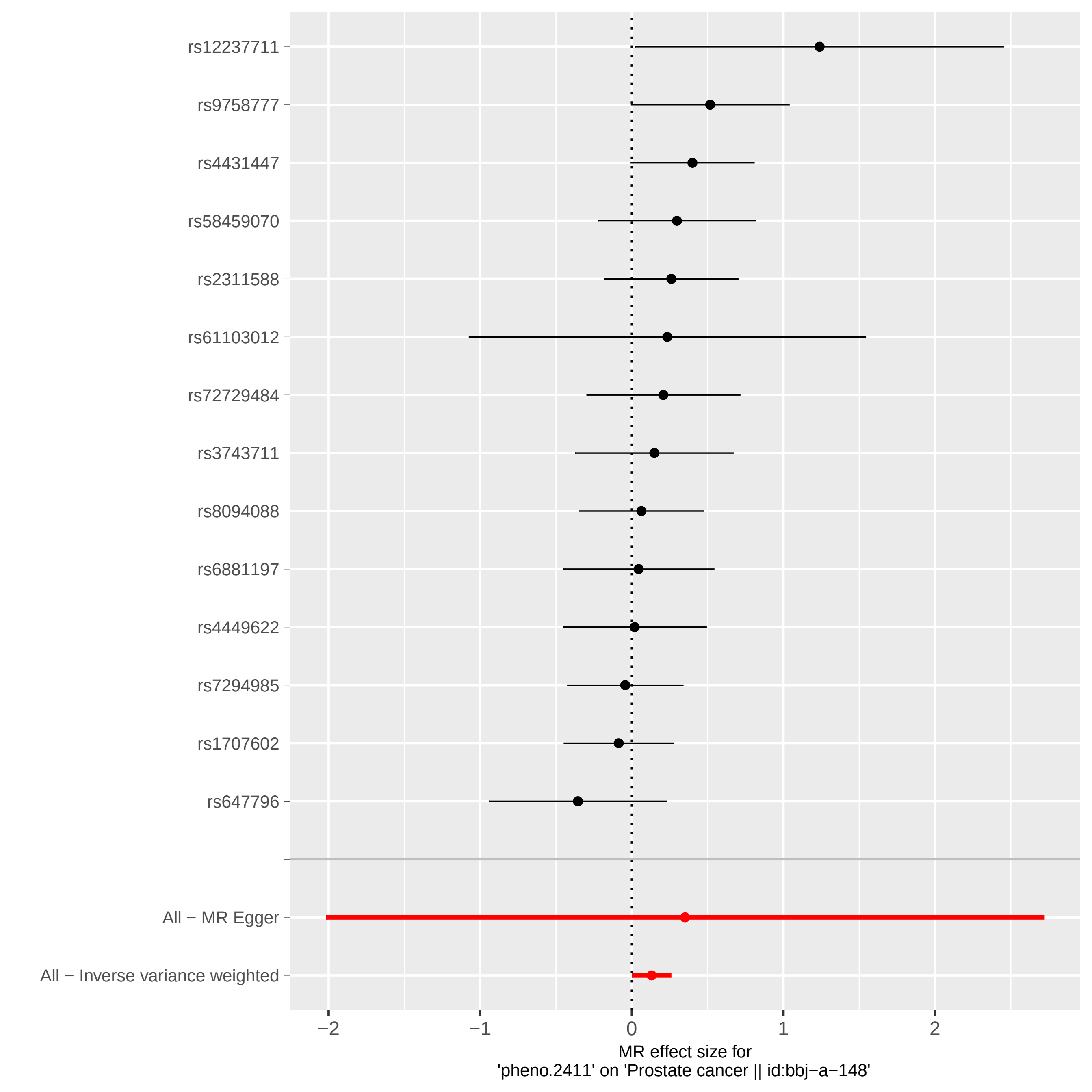
rs12237711
rs9758777
rs4431447
rs58459070
rs2311588
rs61103012
rs72729484
rs3743711
rs8094088
rs6881197
rs4449622
rs7294985
rs1707602
rs647796
All − MR Egger
All − Inverse variance weighted
−2
−1
0
1
2
MR effect size for
'pheno.2411' on 'Prostate cancer || id:bbj−a−148'
